# The efficacy of percutaneous transluminal angioplasty and arteriovenous fistula reconstruction for immature arteriovenous fistula

**DOI:** 10.1186/s12882-023-03361-5

**Published:** 2023-10-17

**Authors:** Han Yu, Yanqing Chi, Baoxing Wang

**Affiliations:** Department of Nephrology, The Third Hospital of Hebei Medical University, 102 Youyi North Street, Qiaoxi District, Hebei province, Shijiazhuang, China

**Keywords:** Percutaneous transluminal angioplasty, Arteriovenous fistula reconstruction, Immature, Vascular access, Patency rate, Hemodialysis

## Abstract

**Background:**

To access the efficacy of percutaneous transluminal angioplasty and arteriovenous fistula reconstruction for immature arteriovenous fistula, compare the long-term patency and post-operative complications between them.

**Materials and methods:**

The medical records and Hemodialysis record sheets from 44 patients between May 2020 and January 2022 who underwent percutaneous transluminal angioplasty or arteriovenous fistula reconstruction treatment for immature autogenous arteriovenous fistula (AVF) were retrospectively reviewed. The patients were divided into two groups according to the type of surgery they received, including 25 patients in the PTA group and 19 patients in the AVF reconstruction group. Clinical outcomes were included, such as the primary and secondary patency rates following the procedure, maturation time, peak systolic velocity (PSV) of brachial artery, maximum pump-controlled blood flow at initial dialysis, and post-operative complications rates in the two groups.

**Results:**

Technical and clinical success was achieved in 100% of the 44 cases. For patients who underwent percutaneous transluminal angioplasty, the primary patency rate at 3, 6, and 9 months was 84.0%, 68.0%, 60.0%, and the secondary patency rate was 92.0%, 84.0%, 80.0%, respectively. And for patients who underwent arteriovenous fistula reconstruction, the primary patency rate at 3, 6, and 9 months was 89.5%, 73.7%, 68.4%, and the secondary patency rate was 100.0%, 94.7%, 94.7%, respectively. There were no significant differences between the two groups in terms of patency rates (*p* > .050). In patients whose maturation was successful, the average maturation time of fistula after the PTA procedure was 19.36 ± 13.94 days, and 58.63 ± 18.95 days for the reconstruction procedure (*p* < .010). The PSV of brachial artery before and after the procedure was 87.64 ± 23.87 cm/s and 153.20 ± 21.69 cm/s in PTA group, for reconstruction group, the number was 86.26 ± 20.59 cm/s and 151.26 ± 29.94 cm/s, respectively. No statistically significant differences (*p* > .050). The maximum pump-controlled blood flow at initial dialysis was 232.60 ± 16.72 ml/min in PTA group, which was significantly higher than 197.11 ± 10.45 ml/min in reconstruction group (*p* < .010). Subcutaneous hematoma, restenosis, thrombus formation, and pseudoaneurysm were major complications in PTA group. Restenosis, thrombus formation, and pseudoaneurysm were major complications in reconstruction group, with no statistically significant differences between the two groups (*p* > .050).

**Conclusion:**

When immature AVFs require reconstruction surgery, the patency outcomes are comparable to AVFs that undergo successful management by PTA. While, when AVFs are successfully managed by PTA, they have significantly less maturation times and higher maximum pump-controlled blood flow rates at initial dialysis AVF use.

## Background

The Arteriovenous fistula (AVF) still remains the gold standard vascular access for patients with end-stage renal disease (ESRD) [1]. AVF maturation is a complex process. The arteriovenous anastomosis is followed by a reactive increase in vessel diameter, and this anastomosis leads to an increase in blood flow, pressure, and vessel wall shear, and exposes the veins to an oxygen-rich environment, promoting a chain reaction of compensatory outward remodeling, lumen expansion, and wall thickening, thereby promoting AVF maturation [1]. The mature AVF is defined as the fistula that can easily puncture during dialysis, have minimal risk of blood leakage during puncture, and can provide sufficient blood flow throughout the dialysis process, but also meet more than 3 times of hemodialysis treatment per week. Vascular ultrasound examination is an important tool to diagnose mature fistula. If achieving the rule of 6s on Duplex Ultrasound (DUS): vein diameter > 6 mm, blood flow > 600ml/min, and vein depth from the skin < 6 mm, we can consider it functional maturation [2]. While for Chinese hemodialysis patients, the rule has changed slightly: vein diameter ≥ 5 mm, blood flow > 500 ml/min, and vein depth from the skin < 6 mm. About 23–46% of AVFs exhibit maturation issues with problems developing adequate vessel diameter and flow for dialysis [1]. A large randomized controlled trial reported about 60% primary maturation failure rate observed with AVFs created using the open surgical technique [3].

The immature AVF is defined as the fistula that has been created within three months but cannot meet the requirements of dialysis [4]. The main components include difficulty in puncture and/or insufficient blood flow. Generally speaking, evaluation of AVF maturation should begin within 6 weeks of the procedure. Patients with long-term kidney disease usually have combinations such as hypertension, and diabetes, which contribute to vascular calcification and the degeneration of artery compliance. Therefore, stenosis in the arteriovenous circuit of a newly constructed AVF is the main reason for failure of the AVF to mature.

Currently, the common used clinical treatments for immature AVF include: Proximal AV neo-anastomosis (PNA), endovascular accessory vein ligation/occlusion, percutaneous transluminal angioplasty (PTA), stent graft placement in long segment stenosed veins [5] and arteriovenous fistula reconstruction, et al. In recent decades, endovascular and surgical procedures have been advocated to promote fistula maturation and increase the number of functional vascular accesses. Percutaneous transluminal angioplasty (PTA) and arteriovenous fistula reconstruction are two main approaches to address stenosis, promoting assisted AVF maturation. To our knowledge, there are no studies directly comparing the effects of PTA and reconstructive surgery on immature arteriovenous fistulas. This article aims to access the efficacy of these two operations, compare the long-term patency and post-operative complications between them, assisting the operator to choose a more appropriate and efficient procedure to increase the percentage of mature arteriovenous fistulas and improve its patency.

## Patients and methods

### Selection of patients

A retrospective review was conducted on 44 patients who had undergone percutaneous transluminal angioplasty or arteriovenous fistula reconstruction treatment due to immature arteriovenous fistula from May 2020 to January 2022, who visited the Third Hospital of Hebei Medical University. Basic clinical data, including, age, gender, primary diseases causing chronic renal failure: Hypertension, Diabetes mellitus, central venous catheter, age of fistula, flow volume, vessels diameters before initial AVF, vessel diameter at the stenosis, degree of stenosis, type of stenosis, were obtained from the electronic medical records and hemodialysis record sheets. The functional status of AVF was obtained through telephone follow-up and outpatient clinic visits to patients. All procedures were in accordance with the principles of the Declaration of Helsinki. The project was approved by the research ethics committee of the Third Hospital of Hebei Medical University (protocol number: K2020-029-1). No written informed consent was required because of the retrospective design of this study.

**Fig. 1:**
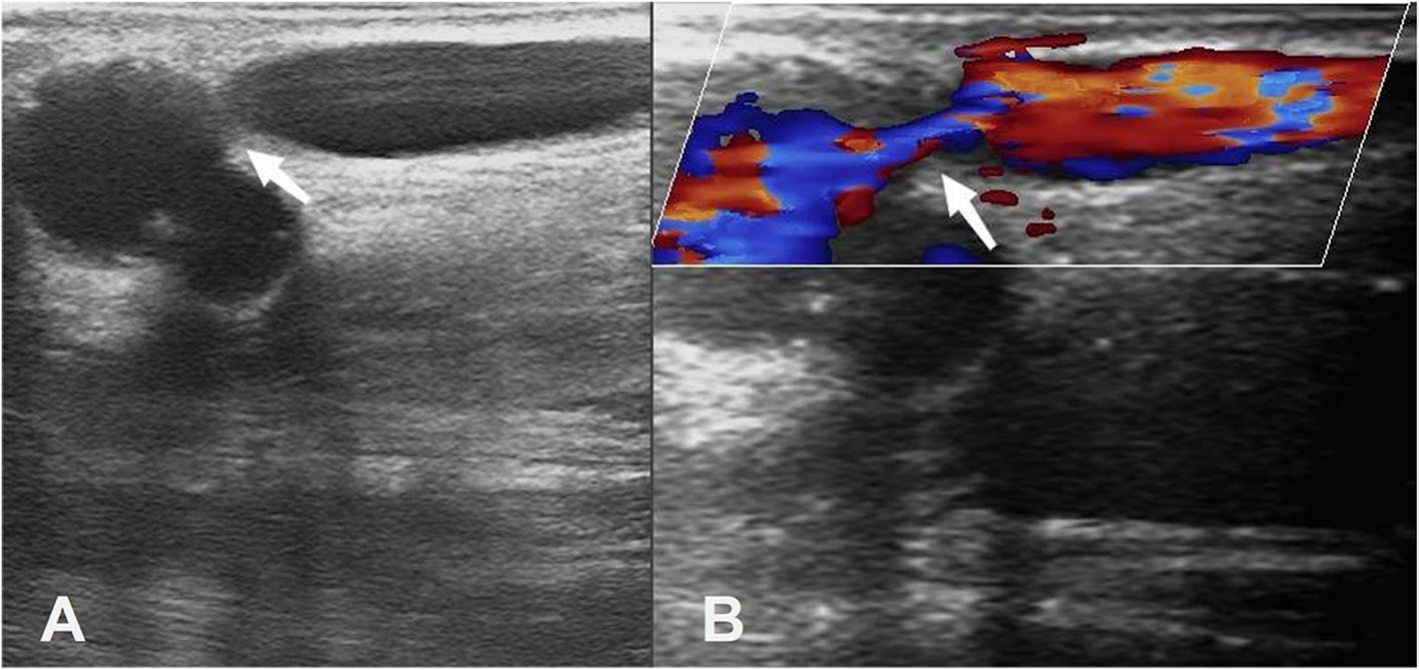
Ultrasound images of immature AVF. Abbreviation: AVF, Arteriovenous Fistula. Note: (**A**) Ultrasound shows significant narrowing of the venous outflow segment (arrow); (**B**) Color Doppler flow image shows significant narrowing of the venous outflow segment (arrow)

Inclusion criteria: (1) Age between 18 and 75 years. (2) Arteriovenous fistula was established less than 3 months ago and did not meet the maturity criteria assessed by Duplex Ultrasound (Fig. 1). (3) Patients with good cognitive function and informed about the research project.

Exclusion criteria: (1) Patients Combined with significant cardiac failure and has not been corrected. (2) Patients with forearm artery malformation. (3) Patients on anticoagulant and antiplatelet therapies or with coagulation system abnormalities. (4) Patients with poor adherence. (5) Patients loss to follow-up or missing information.

The main evaluation indicators of this study include: Primary and secondary patency rates at 3, 6 and 9 months, maturation time, post-operative complications and regular dialysis situations. Fistula patency was observed via physical examination and duplex ultrasonography.

### Treatment procedure

#### Arteriovenous fistula reconstruction

The patient was placed in a supine position with the left/right upper extremity abducted. After routine forearm disinfection, sheeting, and successful local anesthesia with 2% lidocaine. A longitudinal incision was made 5 cm proximal to the stenosis, Subcutaneous tissues were bluntly separated, and the cephalic vein and radial artery were fully exposed. The distal end of the cephalic vein was ligated, and the proximal end was anastomosed with the radial artery end-to-side with continuous external sutures. The Arterial clamp was released, the blood flow through the anastomosis was observed to be smooth, no bleeding and tremor could be palpated, and sutures were closed layer by layer. The subcutaneous tissues and skin were sutured layer by layer and dressed with a sterile dressing. The patient did not complain of discomfort during and after the operation. The anastomotic site was free of blood leakage.

#### Percutaneous transluminal angioplasty

**Fig. 2:**
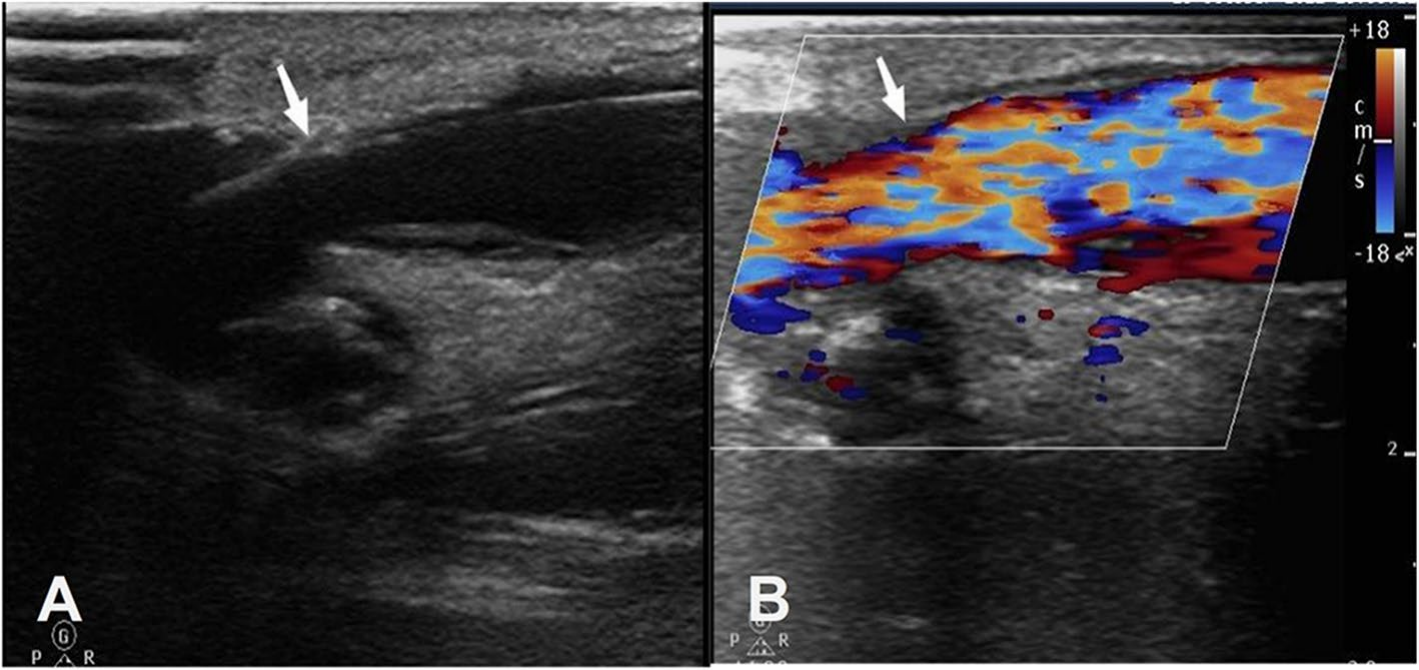
Ultrasound images of the vessel after PTA procedure. Abbreviation: PTA, Percutaneous Transluminal Angioplasty. Note: (**A**) Ultrasound shows an increase in the lumen diameter of the original stenosis after balloon dilation (arrow); (**B**) Color Doppler flow image shows adequate blood flow in the original stenosis segment (arrow)

The patient was placed in a supine position with the upper extremity on the side of the AVF abducted. Routinely disinfected, sheeted, and anesthetized with local infiltration of 2% lidocaine, then the vascular sheath (China, SCW Medicath Ltd. Catalog Number: SCW-IS-0711) was inserted by selecting a suitable access route under ultrasound guidance. Introduce a hydrophilic guide wire (America, Merit Medical Systems, Inc. Catalog Number: LWSTDA35150) along the vascular sheath and then a balloon catheter (China, Bartymedical, Inc. Catalog Number: G0400070004035D) along the guidewire. The diameter of the selected balloon is matched to the diameter of the normal vein adjacent to the stenotic vessel, and the normal balloon is maintained at 8–14 atm, while the high-pressure balloon is maintained at 16–24 atm. The stenotic site is pre-dilated with low pressure first, and then slowly pressurized, repeated 2–3 times, each time for 30–40 s, until the “peak waist sign” at the site of the lesion completely disappears. Use ultrasound to detect the vascular condition again. The results showed that the internal fistula was flowing well (Fig. 2). The balloon, guidewire and vascular sheath were withdrawn after the results showed the patency of the endovascular flow and enhanced local tremor on palpation. The sheath was withdrawn and a tourniquet was applied to stop the bleeding.

Both procedures were performed by the same physician from the Department of Nephrology of the Third Hospital of Hebei Medical University. After the surgery, all the patients were advised to avoid any blood sampling, use of this arm to measure blood pressure, wearing tight clothing or heavy jewelry, lifting heavy objects and compressing the affected limb etc. Patients were instructed to perform functional exercises for one week after discharge from the hospital. At the first post-operative dialysis, instruct the nurse to slowly adjust the pump-controlled blood flow rate of the dialysis machine from 180 ml/min until the dialysis machine alarmed, the dialysis line pumped, microbubbles were generated in the arterial pot, and the absolute value of the arterial pressure before the pump was measured to increase, and then stop. Record the value, which was the maximum pump-controlled blood flow rate that the patient could tolerate. Then slowly adjust the pump-controlled blood flow downward until the patient’s symptoms disappear and the blood pressure returns to normal level. Fistula’s function was obtained at 3, 6, and 9 months through outpatient follow-up visits.

### Definitions and classifications

Technical success is defined as the restoration of blood flow combined with less than 30% residual diameter stenosis. Clinical success is defined as at least one successful dialysis using the AVF after treatment [6]. Primary patency is defined as the time between vascular access creation and the first reintervention due to AVF dysfunction or thrombosis. Secondary patency is defined as the time between one or more interventions and the abandonment of this vascular access [7]. Patency time is defined as the interval between the procedure and the first puncture for dialysis. According to the anatomical site of stenosis, the immature AVF can be divided into 3 types [1]: type I: the juxta-anastomotic area; type II: the outflow vein, especially in the hemodialysis punctured sites; type III: the venous confluence area or central vein, especially in the cephalic-axillary vein confluence or the subclavian-innominate vein confluence.

### Statistical analysis

Establish a database of patient information. The software SPSS, version 22.0 (an IBM Company, Chicago, Illinois, USA), was used for statistical analysis. Continuous variables with normal distribution are expressed as means ± standard deviation (SD). Independent sample T-test was used to compare data between different treatment groups, and paired sample T-test was used to compare data before and after surgery in the same treatment group. Categorical variables are shown as the percentage of patients per group. All the categorical variables were analyzed with the Chi-squared test. The Kaplan–Meier method was used for analysis of patency survival rates. The Graphpad Prism9 was used to draw the survival curves. A *p*-value < 0.050 was defined as statistically significant.

## Results

### Baseline characteristics of the study participants

The baseline characteristics of the 44 study participants were summarized in Table 1. The PTA group included 25 cases, and the fistula reconstruction group included 19 cases. There were no statistically significant differences between the two groups in terms of age, gender, Hypertension, Diabetes mellitus, central venous catheter, age of fistula (Table 1). And the clinical data of the 44 study participants before procedure were summarized in Table 2. In the present study, there were 11 cases of type I stenosis, 6 cases of type II, 7 cases of type I combined with type II, and 1 case of type I combined with type III in the PTA group. There were 13 cases of type I stenosis, 5 cases of type II, 1 case of type III in the fistula reconstruction group. Duplex ultrasound was applied to measure the parameters of the fistulas. The vessel diameter at the stenosis was an average of 0.20 ± 0.06 cm in the PTA group, and 0.18 ± 0.05 cm in the fistula reconstruction group, the difference between the two groups was not statistically significant (*p* = .18). The mean degree of stenosis was 0.61 ± 0.16 in the PTA group and 0.54 ± 0.17 in the AVF reconstruction group, with no statistically significant difference between the two groups (*p* = .16).

**Table 1:** Baseline characteristics of the patients

Groups	PTA group (*n* = 25)	AVF reconstruction group (*n* = 19)	t/ X²-Value	*p*-Value
Age-y	57.9 ± 12.6	62.9 ± 9.4	1.454	.15
Gender (male/female)	15/10	8/11	1.386	.24
Hypertension	23	16	0.650	.42
Diabetes mellitus	14	10	0.049	.82
Central Venous Catheter	18	13	0.066	.80
Age of Fistula-days	56.6 ± 20.5	54.6 ± 27.2	-0.268	.79
Cephalic vein diameter-mm	1.94 ± 0.28	1.96 ± 0.22	-0.278	.78
Radial artery diameter-mm	1.82 ± 0.26	1.92 ± 0.21	-1.444	.16
Systolic blood flow of the radial artery-ml/min	62.08 ± 10.05	65.85 ± 9.54	-1.260	.22
Diastolic blood flow of the radial artery-ml/min	14.77 ± 3.09	15.56 ± 3.32	-0.815	.42

*Abbreviations*: *PTA *Percutaneous Transluminal Angioplasty *AVF *Arteriovenous Fistula

**Table 2:** Clinical data of the 44 cases before procedure

Type of stenosis Groups	Type I	Type II	Type III	Type I + II	Type I + III	Mean vessel diameter at the stenosis	Degree of stenosis
PTA group (*n* = 25)	11	6	0	7	1	0.20 ± 0.06	0.61 ± 0.16
AVF reconstruction group (*n* = 19)	13	5	1	0	0	0.18 ± 0.05	0.54 ± 0.17
t/ X²-Value	8.599					-1.352	-1.418
*p*-Value	0.072					0.18	0.16

*Abbreviations*: *PTA* Percutaneous Transluminal Angioplasty, *AVF *Arteriovenous Fistula. Note: Type I: Stenosis of anastomosis and the cephalic vein within 2 cm of the anastomosis; Type II: Stenosis of puncture segment; Type III: Stenosis of the cephalic vein drains into the axillary vein

### Comparison between the two groups

**Fig. 3:**
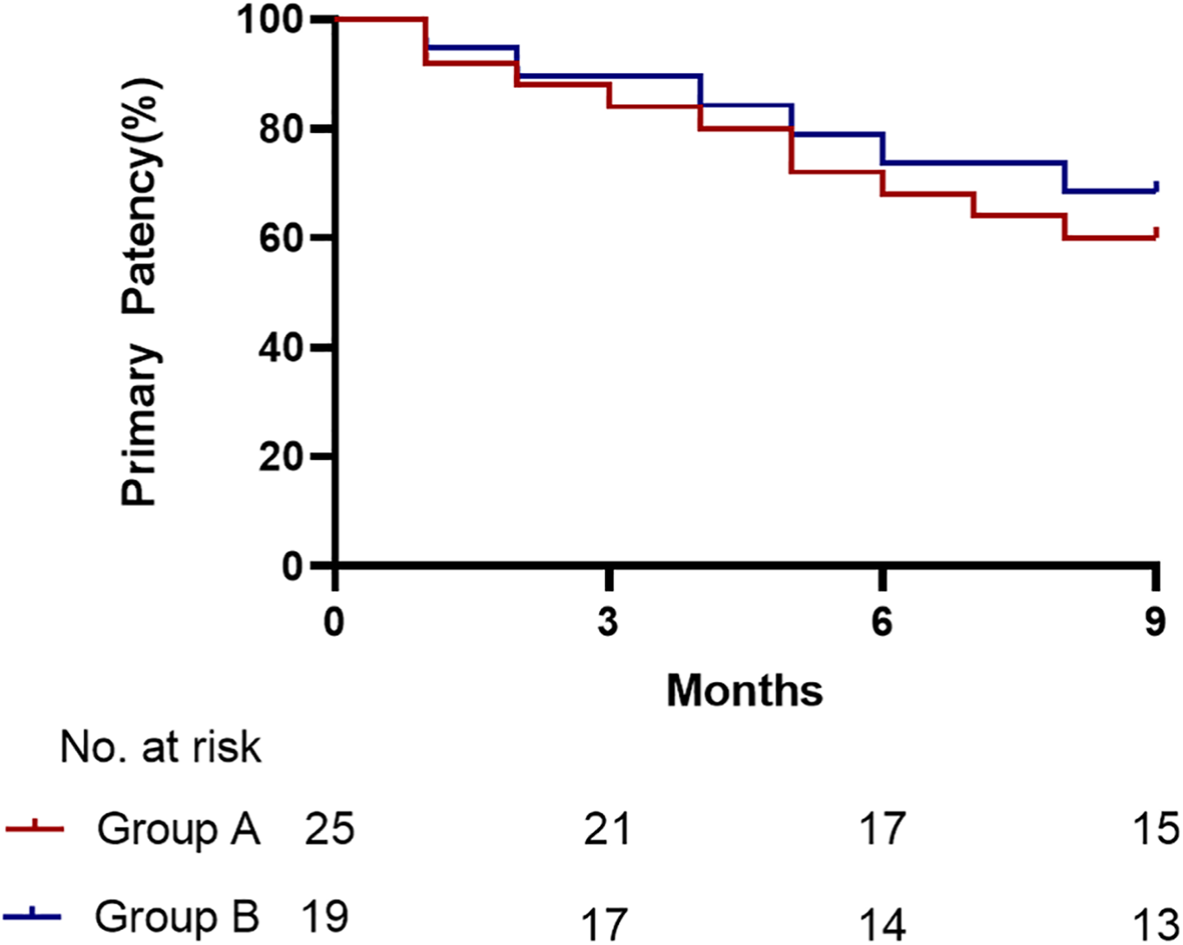
Primary patency after operations. Note: Group A: Percutaneous transluminal angioplasty; Group B: Arteriovenous fistula reconstruction

**Fig. 4:**
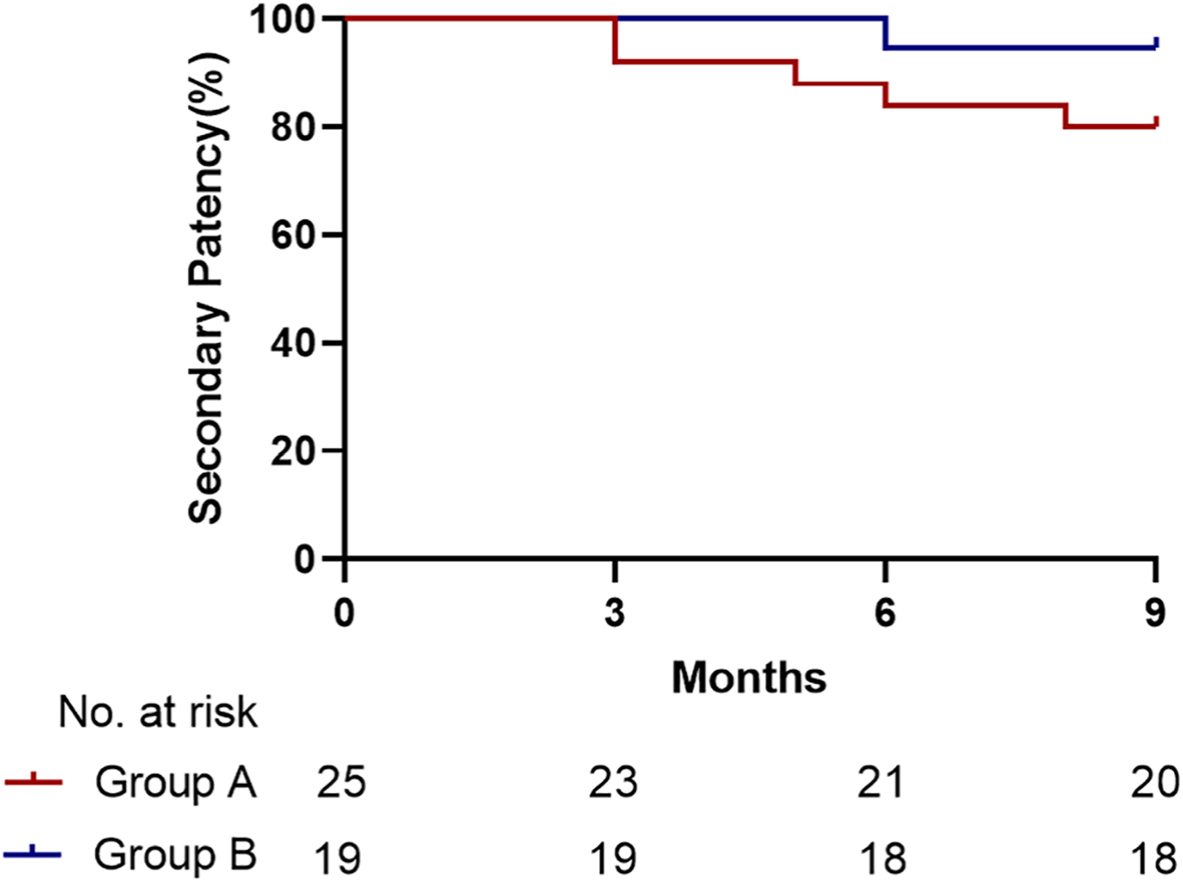
Secondary patency after operations. Note: Group A: Percutaneous transluminal angioplasty; Group B: Arteriovenous fistula reconstruction

All the 44 participants achieved technical and clinical success. The pre-operative average peak systolic velocity (PSV) was 87.64±23.87 cm/s and 153.20±21.69 cm/s on the first postoperative day in the PTA group (*p*=000). For AVF reconstruction group, the results were 86.26±20.59 cm/s and 151.26±29.94 cm/s, respectively (*p*=.000). The results above suggest that both PTA and reconstruction surgery can enhance PSV of brachial artery (*p*=.000), but there is no statistically significant difference between the two groups *(p>*.050，Table 3). The maturation time (the first puncture day for dialysis after operation)  for PTA group and AVF reconstruction group was 19.36±13.94 days and 58.63±18.95 days respectively (*p*<.010). The maximum pump-controlled blood flow rate during hemodialysis for PTA group and AVF reconstruction group was 232.60±16.72 ml/min and 197.11±10.45 ml/min respectively (*p*<.010). For PTA group, primary patency rates at 3, 6, and 9 months were 84.0%, 68.0%, and 60.0%, and secondary patency rates at 3, 6, and 9 months were 92.0%, 84.0%, and 80.0%, respectively. For fistula reconstruction group, primary patency rates at 3, 6, and 9 months were 89.5%, 73.7%, and 68.4%, and secondary patency rates at 3, 6, and 9 months were 100%, 94.7%, and 94.7%, respectively. There were no statistically significant differences between the two groups (Table 4  and Figs. 3 and 4).

**Table 3:** PSV of brachial artery before and after the operation

Groups	PSV of brachial artery-cm/s			
	Pre-operation	Day1 after operation	t-Value	*p*-Value
PTA group (*n* = 25)	87.64 ± 23.87	153.20 ± 21.69	-12.765	0.000
AVF reconstruction group (*n* = 19)	86.26 ± 20.59	151.26 ± 29.94	-8.614	0.000
t-Value	-0.021	-0.238		
*p*-Value	0.84	0.81		

*Abbreviations*: *PTA*  Percutaneous Transluminal Angioplasty,  *AVF *Arteriovenous Fistula, *PSV  *Peak Systolic Velocity

**Table 4:** Maturation time, blood flow and patency rate during the follow-up period

Groups	Maturation Time -days	The maximum pump-controlled blood flow rate -ml/min	Primary Patency Rate			Secondary Patency Rate		
			3 months	6 months	9 months	3 months	6 months	9 months
PTA group (*n* = 25)	19.36 ± 13.94	232.60 ± 16.72	21/25 (84.0%)	17/25 (68.0%)	15/25 (60.0%)	23/25 (92.0%)	21/25 (84.0%)	20/25 (80.0%)
AVF reconstruction group (*n* = 19)	58.63 ± 18.95	197.11 ± 10.45	17/19 (89.5%)	14/19 (73.7%)	13/19 (68.4%)	19/19 (100%)	18/19 (94.7%)	18/19 (94.7%)
t/ X²-Value	7.926	-8.116	0.274	0.168	0.331	1.592	1.236	1.991
*p*-Value	< 0.010	< 0.010	0.60	0.68	0.57	0.21	0.27	0.16

*Abbreviations*: *PTA* Percutaneous Transluminal Angioplasty,  *AVF *Arteriovenous Fistula

### Comparison of complications between the two groups

Among the 44 participants, major complications occurred in 15 patients (34.1%) with subcutaneous hematoma, restenosis, thrombus formation and pseudoaneurysm formation (Table 5). All the participants had no major complications or deaths in our study. 1 patient in the PTA group had a subcutaneous hematoma. Local compression was performed to resolve the hematoma, which did not expand further. 3 patients in PTA group occurred restenosis of venous outflow 2-3 months after surgery. 2 of these three cases were managed successfully with PTA again. There were 3 patients in PTA group and 5 patients in AVF reconstruction group suffered from thrombus formation. Physical compression and urokinase thrombolysis were used to resolve acute thrombosis. Fogarty catheter thrombectomy was used to resolve old thrombosis. There was both 1 patient with pseudoaneurysm in these two groups and was excised by surgery.

**Table 5:** Postoperative complications between two groups

Groups	Subcutaneous Hematoma	Restenosis	Thrombus Formation	Aneurysm/Pseudoaneurysm Formation
PTA group (*n* = 25)	1	3	3	1
AVF reconstruction group (*n* = 19)	0	1	5	1
X^2^-Value	0.778	0.593	1.487	0.040
*p*-Value	0.38	0.44	0.22	0.84

*Abbreviations*: *PTA* Percutaneous Transluminal Angioplasty,  *AVF *Arteriovenous Fistula

## Discussion

Venous stenosis occurs in the vast majority of immature AVFs, whereas, intimal hyperplasia (IH) and vascular fibrosis had been cited the dominating contributors to venous stenosis. The pathophysiological mechanisms of intimal hyperplasia include surgical, pinprick injuries to endothelial cells, hemodynamic alterations, immune or metabolic stresses, which trigger severe downstream responses such as inflammatory cell adhesion, proliferation and migration of smooth muscle cells from the media to the intima [8]. After the arteriovenous anastomosis, the local hemodynamics change significantly, the stress generated by a large amount of blood flow against the vessel wall is called wall shear stress (WSS), it contains two kinds of unidirectional laminal WSS and oscillatory WSS. In the mature AVFs, the impact of high-velocity blood flow produces WSS in the physiological range of the vessel wall, endothelial cells are relatively quiescent and aligned in the direction of blood flow, and the vessel undergoes benign outward remodeling, swelling with some degree of elongation and curvature to accommodate the hemodynamic changes. However, as time passes, blood flow tends to form turbulence in the anastomotic and para-anastomotic venous segments, producing a disturbed, low WSS. Numerous studies have shown that WSS levels are strongly negatively correlated with endothelial hyperplasia. LAN JIA et al. [9] constructed a three-dimensional models of Haemodynamics, showing that significant intimal hyperplasia occurred in the inner wall of juxta-anastomotic segments exposed to low-and-disturbed WSS. The presence of low shear stress promotes endothelial genes and proteins expression of vascular smooth muscle cells (VSMC) mitogens, resulting in intimal hyperplasia [9]. In addition, the oscillating WSS leads to increased expression of chemokines and adhesion molecules and decreased production of vasodilators, inhibits the pathway of NO production and release of matrix metalloproteinases (MMPs), resulting in vasoconstriction rather than vasodilation [10, 11]. Reduced WSS, impaired blood inflow leading to poorly developed venous ducts, and inadequate outflow may also increase vascular resistance, further reducing peripheral blood volume and ultimately leading to poor maturation of newly established AVFs [6].

Mineralocorticoid receptor (MR) is a nuclear receptor and transcription factor that is significantly expressed in the distal renal tubules. MR in endothelial cells, VSMC, macrophages is associated with cardiovascular disease, collectively promoting vascular inflammation, VSMC activation and ECM accumulation [11]. MR activated by mineralocorticoid hormone such as aldosterone triggers non-genomic effects, including increased phosphorylation of GSK3β, c-Src, mitogen-activated protein kinase, and delayed genomic effects, including transcriptional upregulation of placental growth factor signaling pathways and oxidative stress signaling channels via vascular endothelial growth factor type 1 receptors and endothelin [12]. Furthermore, miR-29b in VSMC was found to have a protective effect on vascular remodeling. Activated MR reduced the abundance and activity of miR-29b [13]. All these pathways act synergistically to activate VSMC and enhance its migration, proliferation, and ECM production, which leads to intima-media thickness, luminal narrowing, and vascular fibrosis [12], and eventually contributes to vascular stenosis and immature arteriovenous fistula.

Most stenosis-related immature fistula problems can be solved by endovascular techniques. PTA is an effective intervention procedure for salvaging immature AVFs. Cai et al. [14] established a clinical model of venous stenosis in mice with AVFs. The results showed that after PTA treatment, the levels of endothelial cell CD31, α-smooth muscle cell actin (α-SMA), and cell proliferation (Ki-67) were significantly reduced, and the mean area and cell density of the nascent endothelium and mesothelium were significantly decreased, the luminal vessel area and shear stress increased. Multiple dilation of the stenotic segment using a larger diameter balloon ruptures the intima, media, and adventitia，promoting controlled rupture of the vessel wall or intramural hematoma and exerts sufficient pressure on the vein wall. This results in significant wall dilatation and increased blood flow, reducing the occurrence of perioperative contractures and ultimately creating a large-diameter, well-patented dialysis line [15]. Compared to AVF reconstruction surgery, PTA greatly reduces the maturation time, allowing patients to undergo hemodialysis treatment even within hours or days after the procedure, decreasing the use of temporary catheters, risk of infection and hospitalization costs, effectively protects vascular resources. PTA is often used in conjunction with coil embolization of accessory vessels to improve vascular stenosis and promote AVFs maturation.

In terms of safety, one patient in the PTA group presented with subcutaneous hematoma, three patients with restenosis in venous outflow, three patients with thrombus formation, and one patient with pseudoaneurysm formation. In the AVF reconstruction group, there were one patient presented with restenosis, five patients with thrombus formation, and one patient with pseudoaneurysm formation. In our study, three patients in the PTA group developed restenosis and underwent PTA again 2-3 months after surgery. Rupture of the internal elastic layer is an important trigger for VSMC proliferation and migration [16]. We presume that angioplasty causes mechanical trauma to the vessel with irregular endothelial tears, giving rise to reactive intimal hyperplasia and eventually restenosis.

Systematic review and meta-analysis reported a clinical success rate of 76.2%-96.6% and a technical success rate of 89%-99% for PTA [17]. Study by Leonardo et al. [15] followed up patients accepted PTA up to 12 months with primary patency rate of 87.3%, 66.2%, and 50.7% at 3, 6, and 12 months, respectively, and secondary patency of 100%, 92.9%, and 90.0% at 3, 6, and 12 months, respectively. Our study achieved better expected results, for PTA group, primary patency rates at 3, 6, and 9 months were 84.0%, 68.0%, and 60.0%, and secondary patency rates at 3, 6, and 9 months were 92.0%, 84.0%, and 80.0%, respectively. Study shows that women generally have smaller average blood vessel diameters than men [18], which may lead to a longer time to maturation of AVF and an increased chance of reduced patency. We presume that the poor patency rates were related to the inferior vascular condition in part of female patients, as well as the vascular calcification in elderly people with long-term comorbid chronic diseases such as diabetes and hypertension.

Endovascular treatment is the preferred method for immature AVF due to stenosis. PTA opens up blood flow to the stenosis and restores access function, which not only protects the vascular resources of patients with chronic kidney disease (CKD), but also shortens the maturation time, helping patients with CKD enter the renal replacement therapy stage timely. However, the damage caused to the vessel wall and the economic burden of repeated surgeries should not be underestimated. A variety of safer and more effective alternative materials have been developed to address these problems in clinical practice. Drug-coated balloon (DCB) has been shown to reduce the risk of restenosis. Paclitaxel is the main active component of the coating and has a strong antiproliferative effect. It binds to the b-tubulin, inhibiting the propagation and migration of arterial smooth muscle cells as well as intimal hyperplasia, reducing the inflammatory response of the vessel wall [19, 20]. Primary balloon angioplasty (PBA) of small veins during AVF creation [21], standard (high/ultrahigh) and modified (cutting blades) angioplasty balloon are also gradually being used to improve fistula maturation in clinical practice. Selective coil embolization of accessory vessels [22], the use of VasQ (Laminate Medical Technologies, Israel) device [23], stent graft placement, Clopidogrel and Cilostazol were reported to reduce early fistula thrombosis [3, 24]. Far infrared therapy (FIR) is a non-invasive treatment to promote maturation and patency of AVFs. It produces a thermal effect by transferring heat energy, causing vasodilation and increased access flow. In addition, the non-thermal effect mediated by HO-1 inhibits intima hyperplasia, reduces oxidative stress and inflammation, improves endothelial function [25]. There are a variety of emerging methods to rescue immature AVFs, since vascular resources are precious in patients with chronic kidney disease, so it is always the concern and efforts of every nephrologist to choose appropriate and effective treatment options based on preserving the original vascular access as much as possible.

This study still has several limitations. First, the included study was retrospective design rather than randomized clinical trial. Patients were nonrandomly assigned to either the PTA group or the reconstruction group. A large number of confounding factors from selection and attrition bias may have influenced the results. Despite we attempted to minimize adverse effects through adjustment, unknown confounders or unmeasured confounders may still undermine the robustness of the results.

## Conclusion

When immature AVFs require reconstruction surgery, the patency outcomes are comparable to AVFs that undergo successful management by PTA. While, when AVFs are successfully managed by PTA, they have significantly less maturation times and higher maximum pump-controlled blood flow rates at initial dialysis AVF use. For the moment, PTA is a secure, efficient, economical and widely respected procedure for immature arteriovenous fistulas. Further prospective, large and randomized researches are required to compare the efficacy of PTA and reconstruction surgery.

## Data Availability

The datasets generated and/or analysed during the current study are not publicly available due to the privacy of individual medical records but are available from the corresponding author on reasonable request.

## Abbreviations

PTA: Percutaneous Transluminal Angioplasty
AVF: Arteriovenous Fistula
PSV: Peak Systolic Velocity
ESRD: End-Stage Renal Disease
CKD: Chronic Kidney Disease
IH: Intimal Hyperplasia
WSS: Wall Shear Stress
VSMC: Vascular Smooth Muscle Cell
MMPs: Matrix Metallo Proteinases
MR: Mineralocorticoid Receptor
ECM: Extracellular Matrix
DCB: Drug-Coated Balloon
PBA: Primary Balloon Angioplasty
FIR: Far Infrared Therapy

## References

[1] Lok CE, Huber TS, Lee T, Shenoy S, Yevzlin AS, Abreo K (2020). KDOQI Clinical Practice Guideline for Vascular Access: 2019 update. Am J Kidney Dis.

[2] Kordzadeh A, Askari A, Hoff M, Smith V, Panayiotopoulos Y (2017). The impact of patient demographics, anatomy, Comorbidities, and Peri-operative Planning on the primary functional maturation of Autogenous Radiocephalic Arteriovenous Fistula. Eur J Vasc Endovasc Surg.

[3] Dember LM, Beck GJ, Allon M, Delmez JA, Dixon BS, Greenberg A (2008). Effect of Clopidogrel on early failure of arteriovenous fistulas for Hemodialysis: a Randomized Controlled Trial. JAMA.

[4] Beathard GA, Arnold P, Fau - Jackson J, Jackson J, Fau - Litchfield T, Litchfield T (2003). Aggressive treatment of early fistula failure. Kidney Int.

[5] Bavare CS, Street TK, Peden EK, Davies MG, Naoum JJ (2017). Stent grafts can convert unusable Upper Arm Arteriovenous Fistulas into a functioning Hemodialysis Access: a retrospective Case Series. Front Surg.

[6] Kim Y, Chung BH, Choi BS, Park CW, Yang CW, Kim YS (2019). Outcome of endovascular salvage of immature hemodialysis arteriovenous fistulas. J Vasc Access.

[7] Tawfik AM, Zidan MH, Salem A, Salem A (2022). A randomized controlled study of early versus standard cannulation of arteriovenous grafts in hemodialysis patients. J Vasc Surg.

[8] Jia L, Wang L, Fau - Wei F, Wei F, Fau - Yu H, Yu H, Fau - Dong H, Dong H, Fau - Wang B, Wang B, Fau - Lu Z (2015). Effects of wall shear stress in venous neointimal hyperplasia of arteriovenous fistulae. Nephrology.

[9] Browne LD, Bashar K, Griffin P, Kavanagh EG, Walsh SR, Walsh MT (2015). The role of Shear stress in Arteriovenous Fistula Maturation and failure: a systematic review. PLoS ONE.

[10] Lehoux S, Castier Y, Fau - Tedgui A, Tedgui A. Molecular mechanisms of the vascular responses to haemodynamic forces. Atherosclerotic Plaque. 2021:49–83. 10.1111/j.1365-2796.2006.01624.x 16594906

[11] DuPont JJ, Jaffe IZ, 30 YEARS OF THE MINERALOCORTICOID RECEPTOR (2017). The role of the mineralocorticoid receptor in the vasculature. J Endocrinol.

[12] Koenig JB, Jaffe IZ (2014). Direct role for smooth muscle cell mineralocorticoid receptors in vascular remodeling: novel mechanisms and clinical implications. Curr Hypertens Rep.

[13] Bretschneider M, Busch B, Mueller D, Nolze A, Schreier B, Gekle M (2016). Activated mineralocorticoid receptor regulates micro-RNA-29b in vascular smooth muscle cells. FASEB J.

[14] Cai CA-O, Zhao C, Kilari S, Sharma AA-O, Singh AK, Simeon ML (2020). Experimental murine arteriovenous fistula model to study restenosis after transluminal angioplasty. Lab Anim.

[15] de Oliveira Harduin LA-O, Guerra JB, Virgini-Magalhães CE, da Costa FS, Vieira BR, Mello RS, et al. Oversized balloon angioplasty for endovascular maturation of arteriovenous fistulae to accelerate cannulation and to decrease the duration of catheter use. J Vasc Access. 2021. 11297298211029558. 10.1177/11297298211029558 34218690

[16] Portugaller RH, Deutschmann H, Deutschmann H, Kalmar Pi Fau (2014). The eternal tale of dialysis access vessels and restenosis: are drug-eluting balloons the solution?. J Vasc Access.

[17] Kanchanasuttirak PA-O, Pitaksantayothin W, Saengprakai W, Kanchanabat B. Systematic review and meta-analysis: efficacy and safety of balloon angioplasty in salvaging non-matured arteriovenous fistulas. J Vasc Access. 2022. 11297298221085440. 10.1177/11297298221085440 35389293

[18] Miller CD, Allon M, Allon M, Robbin Ml Fau (2003). Gender differences in outcomes of arteriovenous fistulas in hemodialysis patients. Kidney Int.

[19] Cao ZA-O, Li J, Zhang T, Zhao K, Zhao J, Yang Y (2020). Comparative effectiveness of drug-coated balloon vs balloon angioplasty for the treatment of Arteriovenous Fistula stenosis: a Meta-analysis. J Endovasc Ther.

[20] Lookstein RA, Haruguchi H, Ouriel K, Weinberg I, Lei L, Cihlar S (2020). Drug-coated balloons for dysfunctional Dialysis arteriovenous fistulas. N Engl J Med.

[21] Chawla A, DiRaimo R, Panetta T (2011). Balloon angioplasty to facilitate autogenous arteriovenous access maturation: a new paradigm for upgrading small-caliber veins, improved function, and surveillance. Semin Vasc Surg.

[22] Nauta LA-O, Voorzaat BM, Rotmans JI, Ghariq E, Urlings T, van der Bogt KEA (2020). Endovascular salvage of non-maturing autogenous arteriovenous fistulas by using angioplasty and competitive vein embolization. J Vasc Access.

[23] Karydis N, Bevis P, Beckitt T, Silverberg D, Halak M, Calder F (2020). An implanted blood vessel support device for arteriovenous fistulas: a Randomized Controlled Trial. Am J Kidney Dis.

[24] Jeon JW, Kim HR, Lee E, Lee JI, Ham YR, Na KR (2021). Effect of cilostazol on arteriovenous fistula in hemodialysis patients. Nefrología (English Edition).

[25] Chen CF, Yang WC, Lin CC (2016). An update of the effect of far infrared therapy on arteriovenous access in end-stage renal disease patients. J Vasc Access.

